# The varying effects of antibiotics on gut microbiota

**DOI:** 10.1186/s13568-021-01274-w

**Published:** 2021-08-16

**Authors:** Lulu Yang, Ousman Bajinka, Pa Omar Jarju, Yurong Tan, Aji Mary Taal, Guven Ozdemir

**Affiliations:** 1 Department of Medical Microbiology, Central South University, Changsha, Hunan China; 2 China-Africa Research Center of Infectious Diseases, School of Basic Medical Sciences, Central South University, Changsha, 410078 Hunan China; 3 School of Medicine and Allied Health Sciences, University of The Gambia, Serrekunda, Gambia; 4 Department of Microbiology, University of Nottingham, Nottingham, UK; 5 Section of Basic and Industrial Microbiology, Department of Biology, Faculty of Science, Ege University, Izmir, Turkey; 6 Department of Microbiology, Xiangya School of Medicine, Central South University, Changsha, 410078 Hunan China

**Keywords:** Gut microbiota, Dysbiosis, Antibiotics, Gut health, Narrow spectrum, Broad spectrum antibiotics

## Abstract

Antibiotics are lifesaving therapeutic drugs that have been used by human for decades. They are used both in the fight against bacterial pathogens for both human and for animal feeding. However, of recent, their effects on the gut microbial compositions and diversities have attracted much attention. Existing literature have established the dysbiosis (reduced diversity) in the gut microbiota in association with antibiotic and antibiotic drug doses. In the light of spelling out the varying effects of antibiotic use on gut microbiota, this review aimed at given an account on the degree of gut microbial alteration caused by common antibiotics. While some common antibiotics are found to destroy the common phyla, other debilitating effects were observed. The effects can be attributed to the mode of mechanism, the class of antibiotic, the degree of resistance of the antibiotic used, the dosage used during the treatment, the route of administration, the pharmacokinetic and pharmacodynamics properties and the spectrum of the antibiotic agent. Health status, stress or the type of diet an individual feeds on could be a great proportion as confounding factors. While it is understood that only the bacterial communities are explored in the quest to establishing the role of gut in health, other gut microbial species are somehow contributing to the dysbiosis status of the gut microbiota. Until now, long term natural fluctuations like diseases outbreaks and mutations of the strain might as well rendered alteration to the gut independent of antibiotic treatments.

## Introduction

Antibiotics have been used for decades to prevent the proliferation of bacterial pathogens and thus treatment of bacterial infections. They are used to improve the efficiency of animal feeds (Looft et al. 2012). However, the rate at which bacteria are developing virulence genes enable them to resist these antibiotic. Of recent, the insightful knowledge indicated overuse of antibiotics as impacting gut health. These come with number of health hazardous consequences. Even though antibiotics are saving millions of lives, they are as well reducing residential bacteria and these bacteria are crucial for a healthy gut (Bajinka et al. 2020a, b). Physicians will prescribe broad spectrum antibiotics, often when the pathogen is unknown. While narrow-spectrum targets only few types of bacteria, the devastating effects on gut’s health is incomparable to that of broad-spectrum (Ghoshal et al. 2016).

With the insight on the role of gut microbiota in health and diseases, factors disrupting the gut microbial compositions and diversities have attracted much scientific interest. Cutting-edge researches have established the dysbiosis in the gut flora and the period of recovery due to the use of antibiotics and other drugs. While antibiotics are much studied, an account on the degree of varying effects on gut microbial alteration caused by non-antibiotics are also studied (Bajinka et al. 2020a, b). The use and or the application of other drugs, orally as medication, use on agriculture, gut recovery and healing are looked into, to give evidence based findings. While some common drugs are found to wash away the common phyla, others debilitating effects were observed up to species level. The period of recovery for the gut microbial composition varies with the interventions, the concentration of the drug used, and the length of treatment (Ghoshal et al. 2016). While it is understood that only the bacterial communities are explored in the quest to establishing the role of gut microbiota in disease and health, the alteration of fungal and phage population study will find answers relevant for clinical application.

Previous studies have reached the consensus on degree of known alteration of gut composition. Furthermore, excessive use of antibiotics is associated with some metabolic disorders. Among these, infants and young children are at an increased risk of asthma and weight gain in adults (Vogt and Finlay 2017; Cox and Blaser 2015). Overuse of antibiotic promotes colonization of *Clostridium difficile*, an opportunistic pathogen causing antibiotic-associated diarrhea (Bajinka et al. 2020a, b; Theriot et al. 2014). It leads to reduction in microbial diversity among intestinal flora (dysbiosis) (Dethlefsen and Relman 2011; Zaura et al. 2015; Rashid et al. 2015). Also it cause reduction in the number of protective species such as *Bifidobacterium* spp. (Stewardson et al. 2015)*.* A great deal of studies have centered on the effects of antibiotics on the gut microbiota. Consequently, antibiotics are found to be causing numerous alterations to the microbial population, thereby inflicting changes to the physiological functions of the host (Qiu et al. 2021).

Microbial balance is disrupted both in short and long term conditions due to antibiotic treatment. This causes massive reductions in the richness and diversity of the microbial communities (Clemente et al. 2012). A case scenario will be clindamycin, metronidazole and ciprofloxacin. All of these are affecting the structure of microbiota for varying length of time (Bäckhed et al. 2012; Devillard et al. 2009). Moreover, after disrupting our microbiota by antibiotics, humans showed varying length of time to recovery. It has been proven that, this recovery is individual-dependent. There are possible effects to our variation in microbiota prior to antibiotic treatment (Zivkovic et al. 2011; Ouwehand et al. 2002).

Antibiotics are inevitable in today’s fight against infectious diseases. However, a high dosage is used in order to be strategic and productive (Bajinka et al. 2020b). One explorative study in humans showed devastating results; β-lactam intravenous therapy consisting of ampicillin, sulbactam and cefazolin do not only disrupt the microbial ecology but also the production of strong metabolites are affected (Bajinka et al. 2020a). The effect was seen on the production of acetyl phosphate and acetyl-coA (Liu et al. 2020a, b). These are involved in major cellular functions (Zivkovic et al. 2011). Delayed gastrointestinal (GI) tract motility due to the affected secondary bile acid and serotonin metabolism in the colon was seen among the mice whose microbiota was depleted by antibiotics (Ouwehand et al. 2002). The antibiotic-treated mice were found to be very susceptible to antibiotic-associated pathogens, *S. Typhimurium* and *C. difficile*. Due to the alteration in mucosal carbohydrate availability, pathogens can expand the gut of the mice (Yoshioka et al. 1983).

**Fig. 1:**
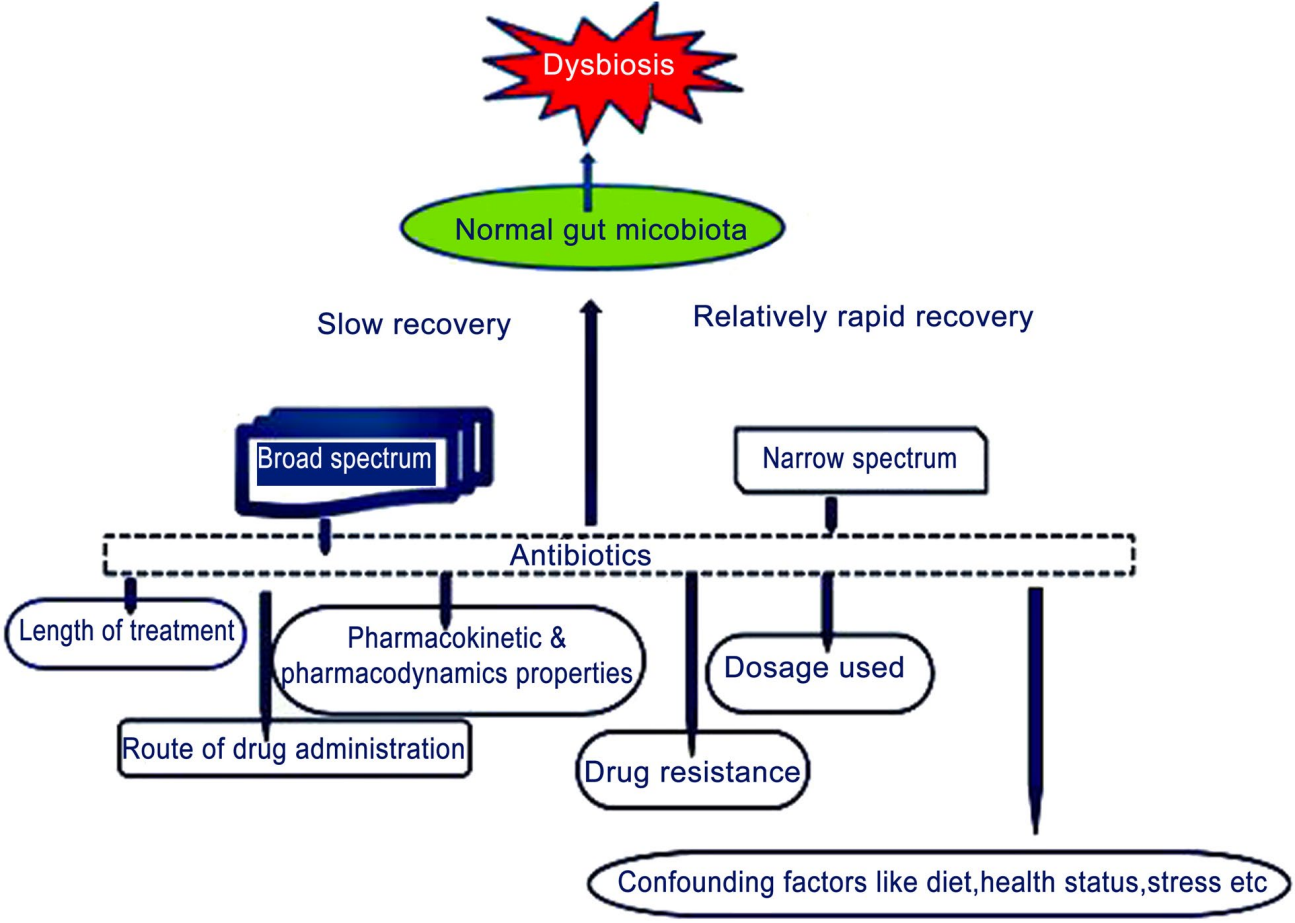
The schematic diagram representing varying effects of antibiotics on gut microbiota. The shorter time drugs are used, the more rapid the recovery of the gut microbiome and vice versa. However, while some will be recovered after months, the extinct species were not seen reinstated during the trial. The proportionate recovery with respect to the concentration of antibiotics per duration is phenomena that will speak in volume as to the efficacy of the used antibiotics

Gene transfer is stimulated among the bacterial population. A reduction in the immune response by the peripheral organs due to the impaired effects is induced through antibiotic intervention (Looft et al. 2012). One such alteration is in the nutritional landscape of the gut. Instead of destroying only the pathogenic microbes, the number of benign microbes will also be reduced (Qiu et al. 2020). *Enterohaemorrhagic E. coli*, *S. Typhimurium* and *C. difficile* all utilize fucose and amino acid liberated by the gut microbiota. For the latter two, an increased level of acid favors healing of their population after antibiotic treatment (Ferreyra et al. 2014; Huang et al. 2015). A schematic illustration shows a hallmark in the use of different forms of antibiotics affecting or inflicting dysbiosis in gut microbiota (Fig. 1).

## Antibiotic abuse

The increase in abuse of antibiotics is undoubtedly causing the increase prevalence of global antibiotics resistance crisis (Liu et al. 2020a, b). While the goal is to receive the correct dosages of the right antibiotics, the cost involved in the medical care resulting from antibiotic abuse during perinatal periods is high (Cardetti et al. 2020). Another form of antimicrobial abuse, which forms the burden of antibiotic use is link to food animal (Adebowale et al. 2020). The genetic capacities of microbes are seen with increasing resistance genes due to overuse of antibiotics. Apparently, bacteria develop multiple mechanisms of resistance against almost all the available antibiotics (Davies and Davies 2010; Munita and Arias 2016). The neurotoxic effect result from abuse of antibiotics works by both local and more prominent systemic mechanisms (Helaly et al. 2019).

The adverse effects of abusing antibiotics include increased microbial resistance and microbiome abnormalities. These abnormalities are called dysbiosis and can lead to autoimmune disorders, gastrointestinal disorders, allergies, infections, arthritis, asthma, cancer and obesity (Cardetti et al. 2020). Abusing antibiotics by taking in excess modifies the gut microbiota and this is studied to be associated with the development of neurological and psychiatric disorders (Helaly et al. 2019). In a bid to cut down the use of antibiotics amid the controlling of abuse of antibiotics and resistance, antimicrobial peptides (AMPs) in combination with some conventional antibiotics are promising (Liu et al. 2020a, b; Pang et al. 2019).

From the recent studies, horizontal gene transfer (HGT) and antibiotic resistance genes happen abundantly in GIT. This has brought number of clinical implications since probiotics, commensals and opportunistic pathogens can survive against antimicrobial medications through transferring resistance genes. Although, gene therapy and personalized medicine could remedy antibiotics resistance, probiotic induced resistant mechanism will require broad interventional study to ascertain the host-specific antibiotic resistant (Daniali et al. 2020).

Since the gut dysbiosis is found to be associated with mucus depletion, inflammation, blood–brain barrier disruption leading to alterations in neuromodulators, patients with drug-resistant epilepsy do suffer from polydrug toxicity (Holmes et al. 2020; Bajinka et al. 2021). A in cohort study of gut microbiota among health individuals in Singapore, high-level tigecycline resistance was detected by the act of tigecycline-inactivating enzyme tet (X4) (tet 4) on host. Tet(X4)-positive isolated from positive Enterobacteriaceae culture resistance mechanism showed that both *E. coli* 2EC1-1 and 94EC carry tet(X4), IncI1-type plasmids p2EC1-1 and p94EC-2 respectively (Ding et al. 2020). Amino acid biosynthesis pathways was found to be required for *Citrobacter rodentium* to colonize the gut in mouse model. However, this pathway is not achievable in antibiotic-treated animals thus exhibiting depletion of amino acids by antibiotics in the gut (Caballero-Flores et al. 2020).

## Use of antibiotics on infant gut and diseases

Gut microbiota is impacted by the early-life antibiotic use. A number of metabolic and immunological diseases are being studied using mouse model. Disrupted microbiome is reflective of the number and types of antibiotic taken. Enough evidence is gathered yet however, the mode of antibiotic delivery is observed to exert different magnitudes of effects (Bajinka et al. 2020a). The Metagenomics, phylogenetic and individual record of used antibiotics were the tools for a study conducted in Finland. Among the children between 2 and 7 years, the use of macrolides was observed to be associated with long-lasting shift in the composition of intestinal flora. This affects the human gut metabolism (Korpela et al. 2016). When children under treatment were compared with non-antibiotic treatment group, a clear difference was observed. Abundances of *Collinsella*, *Lactobacillus* and *Anaerostipes* were seen among non-antibiotics exposed children. These population were reduced among children who were treated with antibiotics. Other alterations include; total richness and maturity of the microbiota remained reduced until after 2 years (Imhann et al. 2016). The recovery is not delayed for all the microbial population. *Bacteroides* and *Bifidobacterium* are observed to recover back to pre-antibiotic status within 12 months after macrolide course. Ironically, children receiving antibiotics registered bacterial infections more frequently than those who do not receive antibiotics. This can be evidence-based on the interrelation and interdependency of the microbes in the gut (Imhann et al. 2016). Wiping a large amount of number is proportionate to weakening the collective functionalities of commensal bacteria.

Mother-to-infant transmission of antibiotic-resistant strains mechanisms are understudied however, health and immune programming of infants is strongly influenced by the types of bacteria that first colonized the gut (vertical transmission). The predicted mechanisms in vertical antibiotic resistant strain transfer from mother to offspring through transfer of AMR strains through breast milk, preterm births and antibiotic exposure. Transfer in utero and during birth (placenta and vagina), through breast milk and transfer of antibiotics in utero (Tochitani 2021). Neonatal antibiotic exposure of cohort of 12,422 children showed weight and height gain among boys and not girls, higher body mass index (BMI) in both gender and a decreased in the diversity of diversity of fecal Bifidobacteria in the first two years of age. This study was follow up by mice model experiment where FMT from children exposed to antibiotics showed growth impairment (Uzan-Yulzari et al. 2021). Again, the expression of genes associated with deficient thalamocortical axons, impaired outgrowth of thalamic axons and axonogenesis were all reduced in a study group of antibiotic-treated mice thus, antibiotic maternal exposure can influence fetal neurodevelopment (Vuong et al. 2020; Patangia et al. 2021). Offspring allergic lung inflammation is strongly associated with prenatal antibiotic exposure through manipulating the neonatal intestinal environment (Alhasan et al. 2020). Ovalbumin-induced anaphylaxis is specifically common among disturbed neonatal immune system development due to exposure to broad-spectrum antibiotic ampicillin. The inability to generate CD4^+^ T cells, colonic regulatory T cells in particular in colons of antibiotic-impacted microbiota mother lead to Treg deficiency. Consequently, this leads to dysregulated Th1 responses when an infant could develop more severe symptoms amends challenges of bacterial infections (Zhang et al. 2021).

## Antibiotic abuse in medical and agricultural purposes

Several studies have concluded that number of drugs disrupt the microbiota, thereby causing gastrointestinal infections. However, not much interventions have been done in response to this. Just as we experience the side effects of some of the orally taken antibiotics, there is clear evidence that gut microbes are as well affected. In this modern agriculture, despite minimal as claimed, doses of antibiotics given to livestock to increase their growth and weight. This is done with the objectives to maximize profit and minimize cost (Looft et al. 2012). In a nutshell, intensive farmers anticipate quick maturity while ensuring healthy livestock for the market consumption (beef and poultry) (Harris et al. 2018). Ironically, we will have beef and poultry for consumption. However, observational human studies, which are coupled with rodent studies, have proven that antibiotics, even a tiny dose found in food cause obesogenic effects for the consumers. Again, to separate the masses and the use of antibiotics require a thorough and global scientific research. Furthermore, the already conducted interventional studies could not engineer consistent metabolic consequences (de Gunzburg et al. 2018). It is hoped that, more carefully designed studies will iron out these kinks.

In swine gut microbiome, bacterial population are found with antibiotic resistome and was as a result of influx treatment of pigs with antibiotics. Although, this enhance pig production, nonredundant genes are now added to reference gene catalogue (RGC) of the pig gut microbiome and these are resistant genes against aminoglycoside and Tetracycline (Wang et al. 2019). Probiotic bacteria are seen with improved conditions for shrimp aquaculture and hence reduction in the proliferation of antibiotic induced-pathogen (Olmos Soto 2021). For instance, lactic acid bacteria of *Enterococcus faecium* Z1-2 and *Enterococcus faecalis* LS1-2 can be used to develop host-specific probiotics (Cui et al. 2017). In a bid to finding alternative to antibiotics, supplementation of rhubarb was found to enhance host mucosal innate immune homeostasis. During the early stages of development, rhubarb could modulate intestinal epithelial microbiota. The gut microbiota was found to shifted in favor of *Clostridium*, *Pseudomonas*, *Blautia*, *Lactobacillus* while a reduction of *Staphylococcus* was observed (Jiao et al. 2018).

## Common antibiotics in use and their effects on gut microbiota

The effects of one antibiotic is severe than the other when administered on the same concentration. One such variation was seen in the study where ciprofloxacin affects rapidly and profoundly lead to loss of gut microbial diversity. A shift in the community compositions just within few days was seen. Notably, individual variation was considered where relevant in the variability of samples. The temporal variation was seen on daily basis that eventually lead to a complete shift. However, there are a number of stable species that resist antibiotic perturbation. Reverting to normal intestinal microbiota after the intervention is often incomplete as some species will never be regained, yet this is different for each individual (Zaura et al. 2015). This can serve as an evidence that human gut microbiota unlike the ecosystem is dynamic in average stable community while non-resilient for all the species. In sense, just as the effect of mutation, once the genes are mutated, there is no recovery. And hence it is imperative to be highly cautious when debilitation our intestinal microbiota with drugs of unknown consequences (Lange et al. 2016; Langdon et al. 2016).

## Clindamycin

Clindamycin is one of the commonly used broad-spectrum antibiotics. It primarily targets anaerobic bacteria, which are vital in maintaining a healthy gut. Clindamycin is excreted in bile and can reach high concentrations in the colon lumen (Vincent and Manges 2015). This disrupts the gut microbiota, causing a shift in colonization by the bacteria. This conditions give rise to the overgrowth of *C. difficile* (Rashid et al. 2015). This in effect, put the gut at risk of pseudo membranous colitis in contrast to the gut of healthy individuals. Diarrhea and gastritis lead to disturbance of normal bowel function thereby bloating. Intestinal pain is prominent and consequently loss of short chain fatty acids (SCFAs). A long term effect was seen on *Bacteriodes* in the colon by a repetitive sequence-based PCR (rep-PCR). Persisting resistant bacteria were observed with an increasing trend (Jernberg et al. 2010).

## Ciprofloxacin versus clindamycin

Studies centered on these drugs have observed a delay in the recovery of normal microbiota for 12 months. The loss or shift in the bacterial composition was observed only after the first month into the intervention. When the two are compared, Clindamycin impacted the gut microbiota greatly more than ciprofloxacin. However, while ciprofloxacin is not associated with the colonization of *C. dificile* (Dethlefsen et al. 2008), Clindamycin is well documented with causing *Clostridium difficile* infection (CDI) (Rashid et al. 2015). When administered orally, gram-negative anaerobic and anaerobic bacteria are not affected by Clindamycin. However, this antibiotic had impacts on gram-negative anaerobes and minor impact on Gram-positive aerobic bacteria (Rashid et al. 2015).

## Amoxicillin

Amoxicillin, when combined with clavulanic acid, exerts devastating effects on the gut microbiome. A total wipe of aerobic gram-positive cocci was observed and an associated increase resistant of enterobacteria when individuals are administered with amoxicillin. Fecal samples of a patient suffering from antibiotic-associated diarrhea were analyzed by 16S rRNA gene clone libraries (Young and Schmidt 2004). Before the initiation of amoxicillin-clavulanate, the gut microbiota comprised mainly of *Bacteriodes*, *Bifidobacterium* and *Clostridium* clusters IV and XIVa (butyrate-producing bacteria). Four days into antibiotic treatment, no sequences corresponding to *Bifidobacterium* and *Clostridium* cluster XIVa was detected. However, a marked increase in *Enterobacteriaceae* was observed. *Bacteriodes* remained consistent throughout the treatment. Prior to antibiotic initiation, *Bacteriodes fragilis* were predominant, whereas by day 4, *Bacteriodes distasonis* became dominant. After cessation of antibiotics, a reversal of the changes was observed, except for *Bifidobacterium*, which did not return. (De La Cochetiere et al. 2005) also observed major changes in dominant bacterial species using temperature gradient gel electrophoresis (TGGE), during and after a 5-day treatment with amoxicillin in six healthy volunteers.

## Clarithromycin

Clarithromycin, metronidazole, and omeprazole all perturb gut microbiota when used in the treatment of *Helicobacter pylori* infections. The effects were observed for both short and long term. At the end of the treatment, *Firmicutes* and *Proteobacteria* dominated the gut environment, however there was a significant reduction in *Actinobacteria*. Jakobsson et al. pyrosequencing data also showed that, these antibiotics also had negative impacts on *Bifidobacterium*. In addition to the reduction of *Bifidobacterium*, another study reported a drastic reduction in *Clostridium* and *Bacteriodes* spp. However, none of the patients was colonized with *C. difficile*. An increase in enterococci was also observed (Adamsson et al. 1999).

## Cefprozil (cephalosporin 2nd generation)

In a specific, reproducible and predictable manner, cefprozil-exposed individuals were seen with an increase *Lacnoclostridium bolteae*. An elevated rate of the occurrence of opportunities pathogens after receiving antibiotic treatment has been observed with lower microbial diversity and profoundly in *Bacteriodes enterotype*. In addition to the alteration in the gut microbiome, a point mutation in beta-lactamase bla CfxA-6 was enriched by the antibiotic when compared with resistant genes prior to the intervention of Cefprozil (Raymond et al. 2016). A summary of the varying effects of antibiotics and antibiotics usage against alteration caused to gut microbiota is worth exploring (Tables 1, 2).

## Imipenem

One of the outstanding performance for imipenem is that, among many common antibiotics, it was found to treat *Escherichia coli* despite, extended-spectrum β-lactamase (ESBL) genotype (Hoang et al. 2017). Imipenem–cilastatin antibiotic use for the treatment of neutropenic fever was found to be associated with graft-versus-host disease (GVHD)-related mortality at 5 years. This treatment brought in loss of protective mucus lining of the colon thereby desrupting the intestinal barrier function with *Akkermansia muciniphila* expansion. However, despite this pathological evidence with imipenem, it does not affect numbers of regulatory T cells and SCFAs (Shono et al. 2016). Fecal samples from intensive care patients showed a novel strain with 99% nucleotide similarities to *Sellimonas intestinalis* BR72T resistant to antibiotics imipenem (Versluis et al. 2019).

## Colistin

A combination of colistin and amoxicillin was found to induced antibiotic resistome and altered gut microbiota. This shift could not be completely recovered to baseline even after FMT treatment (Li et al. 2021). Phenotypic colistin-resistance associated plasmid-mediated mobile colistin resistance (mcr) genes was found very prominent in chicken-gut bacteria. About 61.7% of the samples were found to developed resistance to colistin (Islam et al. 2020). Among the last resort antibiotics is colistin. However, of recent, mcr genes in some common bacteria are posing antibiotic resistant to this antibiotic treatment. In addition to Suterella and Parasuterella, all of the other genus *Escherichia*, *Campylobacter* and *Vibrio* carries mcr-like genes. With the knowledge that in *Sutterella. wadsworthensis* mcr-like genes fused to a PAP2-like domain will lay forward some insights into the genetic mobility and strain-specific colisitn resistant (Andrade et al. 2021). A reduced number of *Phascolarctobacterium succinatutens*, *Prevotella copri*, *Prevotella stercorea* and expansion of *Acidaminococcus fermentans* and *Treponema succinifaciens* in a study of colistin on gut microbiome composition in pig. In addition, the dominant signaling pathway induced by colisitin intervening group were mitogen-activated protein kinase signaling pathway-yeast, various types of N-glycan biosynthesis, pathogenic *Escherichia coli* infection, protein processing in endoplasmic reticulum and oxidative phosphorylation (Guo et al. 2021).

## Prospects

The type of antibiotic, the mode of mechanism and the level of resistance determines how it affects the targeted population and the residential microbes. Besides the type-specific effects or alteration of gut microbiota by specific antibiotics, the dosage and duration of antibiotic treatment, route of administration, the pharmacokinetic and pharmacodynamics properties and the spectrum of the antibiotic agent all confer variable alteration to the gut microbiota. The variation in individual gut response on the baseline antibiotics treatment should as well be considered as confounding factors. These are the health status of the person and stress or the type of diet the individual feeds on predominantly. This will lessen the forms of bias in the clinical trials set to establish the effects of antibiotics on the gut. Consequently, depending on the results gathered from short-term intervention alone might not be enough. In essence, long-term natural fluctuations while empirically data are strictly recorded will add more weight on the anticipated Metadata.

The study of resistant genes prior to treatment and after, will answer questions as to the confounding factors leading to such mutations. In fact, in the absences of one bacterial population, gene transfer seems to be prominent among the other groups and in the effect, giving rise to varying resistant genes. More scholarship attention should be centered on effects of antibiotics of the gut microbiota leading to antibacterial resistance. Unlike bacteria, studies have established that gut virome is stable over 12 to 26 months and even individual specific, meaning there is no ‘core gut virome’. Bacteriophages are seen to exert effects on FMT treatment of CDI (Zuo et al. 2018).

Antibiotics are meant to target the bad bacterial population. However, their chemical composition will alter the interspecies relationship. Furthermore, there are still very limited to based arguments on. Most of the virus living in the human gut are bacteriophages. When considering the principle of antibiotics, it has similar functions with these bacteriophages. However, there is a knowledge gap as to understanding the interaction among the pathogens, the benign bacteria, and the bacteriophages. The study of microbiota is no doubt beyond the characterization of diverse communities; it should encompass the total sum of the dynamic interaction among all the microbes, the host and the external factors. We might say that it is still at the infancy since only bacteria are explored to a larger extent. We still don’t know the viral species co-inhabiting human cells. Since the bacteriophages presentable over time, is not exactly the same with the other person, what phage composition is there to limit the bacterial growth? What phyla of bacteria are affected in order to establish a healthy-like gut is a potential research scholarship. In order to have an insight into what the futures of our gut microbiota with respect to the physiological response to disturbance and the changes that these disturbances might bring in, a baseline temporal variation is required (Zaura et al. 2015).

A baseline of gut virome is exactly what we need to ascertain as to what composition signals a healthy gut. This begs for a broad study that must consider the lifestyle of the cohort at study. The geographical differences and ethnicity since the data will serve as the baseline for the preceding researches. Apparently, the alteration inflicted upon by antimicrobial intervention will be evaluated. Furthermore, knowing what a healthy gut is comprised of, with respect to virome will lead ways to the study of gut-related viral diseases. However, from the emerging data, almost 99% of the gut virus is unknown. To rely on the existing sequence database in establishing the baseline is worth not taken. Assembling the reads into overlapping DNA sequences in a bid to predict protein-coding genes for uniformity is hoped to provide clues as to the type virus.

The interventions that this review will predict in order to reduced the antibiotic induced dysbiosis in the gut are; the use of prophylactic antibiotics should be reduced and devising alternatives to antibiotics during pregnancy. Again, studies should be designed to elucidate on the influence of gut microbiome composition and resistome being affected by antibiotic treatment during pregnancy and lactation. Also the mechanisms in which antibiotics can reach mothers milk and concentrations are enough to affect the breast milk microbiome. It will be prudent enough to know with time, how antibiotic influence microbial diversity and the recovery. Moreover, at strain level, transfer of resistant bacteria in utero should be investigated as to affecting early gut colonization. This should be jointly studied with the risk factors for transmission. Altogether, these could help in establishing healthy gut among mothers and their offsprings.

## Conclusion

In summary, our review showed that both gram-positive and gram-negative bacteria are disrupted with broad-spectrum antibiotics as oppose to narrow spectrum antibiotics. However, what limits interventions to mitigate towards these damages the gut microbiota is our inadequate knowledge in antibiotic activity spectra, the resistant mechanisms of gut microbes against various used antibiotics and the extent of damages specific antibiotics caused. While non antibiotics such as probiotics contribute to microbiome-facilitated antibiotic resistance, the interactions between drugs and microbiome are found to be bidirectional. Antibiotics can disrupt gut microbiota and microbes as well can modulate the drugs thereby modifying the chemical composition and leads to the antimicrobial resistance. Now, our hopes are precision medicine, resistant gene therapy and system biology approaches to leverage the host microbe interaction with an effective and safe treatment strategies.

## Data Availability

Not applicable.

## Annex

See Tables 1, 2.

**Table 1:** The varying effects of antibiotics usage against alteration caused to gut microbiota

Antibiotics	Clinical/RCT use	Alteration in the gut
Amoxicillin and clarithromycin	Used for short term treatment of certain bacterial species, duodenal ulcers	^a^*Firmicutes* and ^b^*Bacteriodetes* (Oh et al. 2016)
Clindamycin	Commonly used in broad based antibiotics for treatment of skin and vaginal infections	^a^*Lactobacilli*, *Clostridia, Bacteroides*, *Bifidobacteria*, *C. dificile* ^b^*Enterococci* and *Enterobacteria* (*Klebsiella*, *Enterobacter* and *Citrobacter*) (Clemente et al. 2012)
Clarythromycin	To treat wide range of infections including skin, respiratory and stomach ulcers caused by *H. pylori*	^a^*Firmicutes*, *Proteobacteria*, *Enterococci*, bile-salt hydrolase (Korpela et al. 2016) ^b^*ActinobacteriaBifidobacteria*, *Actinobacteria*, resistance gene *erm* (B) (Jakobsson et al. 2010)
Cefprozil	To treat wide range of bacterial infections	^a^*Lacnoclostridiumbolteae*and ^b^*Bacteriodes enterotype*
Cefepime	To treat bacterial infections such as, urinary tract infections (UTI), pneumonia and skin infections	^b^*E. coli* and *Bifidobacteria*
Ceftazidime	To treat severe or life-threatening bacterial infections	^b^*Enterobacteriaceae* and *Lactobacilli*
Imipenem	To treat severe infection of heart, lungs, blood, bones, joints, female reproductive organs etc.	^b^*Enterobacteriaceae*, *Clostridia*, *Bacteriodes* and *Enterococci*
Meropenem	To treat infections of the abdomen such as appendicitis and peritonitis, bacterial meningitis infections	^a^*Enterococci*, *Enterobacteriaceae*, *Clostridia* and *Bacteriodes*
Chlortetracycline and sulfamethazine	Use for cattle feeding	^a^*Proteobacteria*, profoundly *E. coli* (Reijnders et al. 2016)
Piperacillin/tazabactam	To treat bacterial pneumonia and skin, also gynecological and abdominal disorders	^b^*Enterobacteriaceae*, *Bifidobacteria*, *Eubacteria* and *Lactobacilli*
Ciprofloxacin	To treat various types of bacterial infections, skin, joint, bone UTIs, sinus and respiratory	^a^*Enterococci*and ^b^*Enterobacteriacea* (Stewardson et al. 2015)
Levoflaxacin	To treat bacterial infection of lungs, ears, airways, bones, sinuses and joints	^b^*E. coli*
Azithromycin Azithromycin + metronidazole	To treat respiratory infections and asthma-like episodes in children	Short-term—^b^*Bifidobacterium* (Wei et al. 2018) Faecal calprotectin declined significantly (Levine et al. 2019)
Amoxicillin–clavulanate (AC)	To treat infections like, Sinusitis, pneumonia, ear infections, UTIs etc.	^a^*Escherichia*, *Parabacteroides*, *Enterobacter* and ^b^*Roseburia* (Kabbani et al. 2017)
Vancomycin, (obese and pre-diabetes men)	To treat infections caused by *C. dificile* and methicillin resistance *Staphylococcus areaus* (MRSA) CDI treatment	^b^*Firmicutes* (Reijnders et al. 2016) ^b^Firmicutes (Cannon et al. 2017)
(Ciprofloxacin, vancomycin and metronidazole) for 7	To test whether disruption of the gut microbiota affects systemic innate immune responses during endotoxemia in healthy subjects	^b^Gut diversity however, no impact on agents of sepsis (*Streptococcus pneumonia, Klebsiella pneumonia*, *E. coli*) (Lankelma et al. 2017)
Surotomycin	To determine the impacts of ascending doses of surotomycin on major organism groups in the gut microbiota CDI treatment	^b^*Clostridia*, *Lactobacillus*-*Bifidobacterium*, *Enterococcus*-*streptococcus* and *Enterobacteria* and *B. fragilis persisted* (Citron et al. 2016) ^a^*Bacteroidetes* and *Prevotella* (Cannon et al. 2017)
Preterm birth + antibiotic	To determine whether the differences in gut microbiota between late preterm and full-term infants results from prematurity or external exposures	Prevalence differences in *Bifidobacteria* in full-term and ^b^diversity late preterm neonates (Bajaj et al. 2018)
Rifaximin (open-label treatment for 2 weeks) Rifaximin [common variable immunodeficiency (CVID) patients]	To explore potential effects of rifaximin on the gastrointestinal microbial community in patients with IBS-D	^b^*Peptostreptococcaceae*, *Verrucomicrobiaceae*, *Enterobacteriaceae* at a 10% false discovery rate threshold (Oh et al. 2016) The gut bacteria in the CVID dysbiosis index were not changed (Jørgensen et al. 2019)
Lactulose and rifaximin) + FMT (*Lacnospiraceae* and *Ruminococcaceae*)	The role of faecal microbial transplant (FMT) in restoring gut microbial function is unclear in cirrhosis	^b^Microbial diversity and autochthonous taxa relative abundance (Harris et al. 2018)
Fluoroquinolone antibiotic moxifloxacin + DAV132	To deliver a powerful adsorbent, activated charcoal, in the late ileum of human volunteers	Preserved gut microbial diversity (de Gunzburg et al. 2018)

^a^Denotes increased in abundance, expansion etc.

^b^Denotes decreased in abundance, reduced etc.

**Table 2:** The varying effects of probiotics and prebiotics, and other used drugs on gut microbiota

Drug	Medicinal use	Alteration in the gut
Proton pump inhibitors (PPIs)	To treat dyspepsia To eradicate *H. pylori* in the treatment of peptic ulcer, for Gastro reflux and Barrett’s esophagus	^a^*Rothia*, *Enterococcus*, *Streptococcus*, *Staphylococcus* and the potentially pathogenic species *Escherichia coli* (Jackson et al. 2016) ^b^Shannon9s diversity
Antidepressants	To balance neurotransmitters	^a^*Eubacterium ramulus*, *Clostridium* ^b^*E. coli*
Laxatives	Treatment and prevention of constipation that affects up to 17% of European adults yearly	^a^*Enterobacteria* and *Streptococcus*. Also, laxatives decrease *Lactobacilli* causing intestinal permeability
Oral steroids	Anti inflammatory therapy	^a^Methanogenic bacteria associated with obesity and also an escalating BMI
Drinking water of Electrochemically reduced alkaline (pH ≈ 9)	To determine electrochemically reduced alkaline (pH ≈ 9) versus neutral (pH ≈ 7) drinking water (2 L/day) on human gut microbiota and host glucose metabolism	^a^*Clostridiales*, family *Ruminococcaceae*, genus *Bacteroides*, and species *Prevotellacopri*
Metformin	Metformin is used to treat Type 2 diabetes	*E. coli* is predominantly seen

Probiotics	Medicinal use	Alteration in the gut
Probiotics *Lactobacillus rhamnosus* GG (5 × 10^9^ cfu)	To ameliorate the effects of antibiotic use or caesarean birth on infant microbiota	Only breastfed infants showed the ^a^*Bifidobacteria* ^b^*Proteobacteria* and *Clostridia* (Korpela et al. 2018)
Auto-FMT during allo-HSCT treatment	To re-establish depleted gut commensal	^a^Microbial diversity and reestablished the intestinal microbiota composition (Taur et al. 2018)
6 g/day prebiotics inulin-type fructans or maltodextrin	To reduce febrile episodes requiring medical attention and to lower the incidence of sinusitis	^a^*Bifidobacterium* (with antibiotics) ^b^*Bifidobacterium* (non-antibiotic) (Soldi et al. 2019)
Probiotics intervention of *Bifidobacterium* spp.	To determine the effects of a Bifidobacteria-containing formula on the healthy human intestinal microbiome during the first year of life	^b^*Bacteroides* and *Blautia* spp. (Bazanella et al. 2017)
Synbiotics therapy	To evaluate whether Synbiotic (pre- and probiotics) therapy alters the gut microbiota	^a^*Bifidobacterium* ^b^*Ruminococcaceae* (Zuo et al. 2018)
Concomitant therapy antibiotic + probiotics regimen	To investigate the effects of a commonly used anti-*Helicobacter pylori* concomitant therapy on the composition of the gut and throat microbiota	Several alteration based on fecal culture however reverted by day 71 with the exception yeast and *Bacteroides* spp. (Wang et al. 2017)
Broad-spectrum (oral vancomycin, ciprofloxacin, metronidazole)	To test if microbiome modulation can improve oral rotavirus vaccine (RVV) immunogenicity	^b^Gut bacterial beta diversity and alters the immune response to RVV (Harris et al. 2018)

^a^Denotes increasing or expansion

^b^Denotes decreasing or reduction in number

## Abbreviations

AC: Amoxicillin–clavulanate
BMI: Body mass index
*CDI*: *Clostridium difficile* infection
CVID: Common variable immunodeficiency
FMT: Faecal microbial transplant
GVHD: Graft-versus-host disease
HGT: Horizontal gene transfer
mrc: Colistin resistance
MRSA: Methicillin resistance *Staphylococcus areaus*
RCT: Randomized controlled trial
rep-PCR: Repetitive sequence-based PCR
RCG: Reference gene catalogue
SCFAs: Short chain fatty acids
TGGE: Temperature gradient gel electrophoresis
tet 4: Tigecycline-inactivating enzyme Tet (X4)
UTI: Urinary tract infections
